# Cervidae antlers exploited to manufacture prehistoric tools and hunting implements as a reliable source of ancient DNA

**DOI:** 10.1016/j.heliyon.2024.e31858

**Published:** 2024-05-24

**Authors:** José-Miguel Tejero, Olivia Cheronet, Pere Gelabert, Brina Zagorc, Esteban Álvarez-Fernández, Pablo Arias, Aline Averbouh, Guy Bar-Oz, Omry Barzilai, Anna Belfer-Cohen, Marjolein D. Bosch, Florian Brück, Marián Cueto, Martin Dockner, Josep Maria Fullola, Diego Gárate, Michael Giannakoulis, Cynthia González, Nino Jakeli, Xavier Mangado, Tengiz Meshveliani, Petr Neruda, Philip Nigst, Roberto Ontañón, Maayan Shemer, Petra G. Šimková, Jesús Tapia, Marta Sánchez de la Torre, Catherine Schwab, Gerhard Weber, Ron Pinhasi

**Affiliations:** a Seminari D'Estudis I Recerques Prehistòriques (SERP), Dep. Història i Arqueologia, University of Barcelona, Spain; b Department of Evolutionary Anthropology, University of Vienna, Austria; c Human Evolution and Archeological Sciences (HEAS), University of Vienna, Austria; d Institut D'Arqueologia de La Universitat de Barcelona (IAUB), Spain; e Departament de Biologia Animal, de Biologia Vegetal I D'Ecologia, Universitat Autònoma de Barcelona, Bellaterra, Spain; f GIR PREHUSAL, Departamento de Prehistoria, H^a^ Antigua y Arqueología, Universidad de Salamanca, Spain; g Instituto Internacional de Investigaciones Prehistóricas de Cantabria (IIIPC), (Universidad de Cantabria-Gobierno de Cantabria-Santander Universidades), Santander, Spain; h CNRS-MNHN UMR 7209 Archéozoologie, Archéobotanique: Sociétés, Pratiques et Environnement. Muséum National D’Histoire Naturelle, Département « Homme et Environnement » & Institut INEE CNRS « Environnement et écologie », Paris, France; i Laboratory of Archaeozoology, School of Archaeology and Maritime Cultures, University of Haifa, Israel; j The Leon Recanati Institute for Maritime Studies, School of Archaeology and Maritime Cultures, University of Haifa, Mount Carmel, 3498838 Haifa, Israel; k Institute of Archaeology, The Hebrew University of Jerusalem, Israel; l Austrian Archaeological Institute – Prehistory Austrian Academy of Sciences, Vienna, Austria; m Departament de Prehistòria, Universitat Autònoma de Barcelona, Spain; n Uppsala University for Applied Sciences, Uppsala, Sweden; o Independent Researcher, Tbilisi, Georgia; p Georgian National Museum, Tbilisi, Georgia; q Moravské Zemské Museum, Historické Muzeum, Ústav Anthropos, Brno, Czech Republic; r Department of Prehistoric and Historical Archaeology, University of Vienna, Austria; s Museo de Prehistoria y Arqueología de Cantabria (MUPAC), Santander, Spain; t Department of Bible, Archaeology and the Ancient Near East, Ben Gurion University of the Negev, P.O. Box 653, Beer Sheva, 84105, Israel; u Sociedad de Ciencias Aranzadi, Donostia, Spain; v Musée D’Archéologie Nationale et Domaine National de Saint-Germain-en-Laye, France

**Keywords:** Ancient DNA, Antler, Upper palaeolithic, Hunting implements, Osseous tools

## Abstract

Antler is one of the primary animal raw materials exploited for technical purposes by the hunter-gatherer groups of the Eurasian Upper Palaeolithic (UP) all over the ecological range of deers, and beyond. It was exhaustively employed to produce one of the most critical tools for the survival of the UP societies: hunting weapons. However, antler implements can be made from diverse deer taxa, with different ecological requirements and ethological behaviours. Identifying the antler's origin at a taxonomic level is thus essential in improving our knowledge of humans' functional, practical and symbolic choices, as well as the human-animal interface during Prehistoric times. Nevertheless, palaeogenetics analyses have focused mainly on bone and teeth, with genetic studies of antler generally focused on modern deer conservation. Here we present the results of the first whole mitochondrial genome ancient DNA (aDNA) analysis by means of in-solution hybridisation capture of antlers from pre-Holocene archaeological contexts. We analysed a set of 50 Palaeolithic and Neolithic (c. 34-8ka) antler and osseous objects from South-Western Europe, Central Europe, South-Western Asia and the Caucasus. We successfully obtained aDNA, allowing us to identify the exploited taxa and demonstrate the archaeological relevance of those finds. Moreover, as most of the antlers were sampled using a minimally-invasive method, further analyses (morphometric, technical, genetic, radiometric and more) remain possible on these objects.

## Significance statement

Antlers from the Cervidae family are one of the most exploited raw materials from the Palaeolithic period. Nevertheless, antler implements can be made from diverse deer taxa with different ecological requirements and ethological behaviours. It is thus essential to know their taxonomic origin to evaluate the synergy between the hunter-gatherers, their prey and their environment. Here, we successfully conducted a whole mitochondrial genome aDNA analysis of antlers by means of in-solution hybridisation capture from pre-Holocene archaeological contexts. We demonstrate that implements made from the antlers of undefined taxa can be attributed to a species, enabling deeper archaeological inferences. Although other methods, like palaeoproteomic analyses, can identify at a family taxonomic level, only aDNA allows us to identify the exploited species and perform further phylogenetic analyses.

## 1 Introduction

Objects made from diverse internal and external skeletal tissues (e.g., bone, antler, ivory, teeth, shell) are one of the most common archaeological remains recovered from prehistoric sites from the Palaeolithic to the most recent periods. Among these various osseous tissues exploited for technical purposes, deer antler is one of the main animal raw materials chosen by Eurasian Upper Palaeolithic (UP) hunter-gatherer groups across the deer's ecological range and beyond. It was exhaustively employed to produce hunting weapons, one of the most important tools for the survival of the UP societies [[1], [2], [3], [4], [5], [6], [7], [8], [9], [10], [11], [12], [13]]. Furthermore, other "domestic" tools, like chisels and awls [[14], [15], [16]], and even mobile art and personal ornaments [[17], [18], [19], [20], [21], [22], [23], [24], [25]], were sometimes also made using antler.

**Fig. 1:**
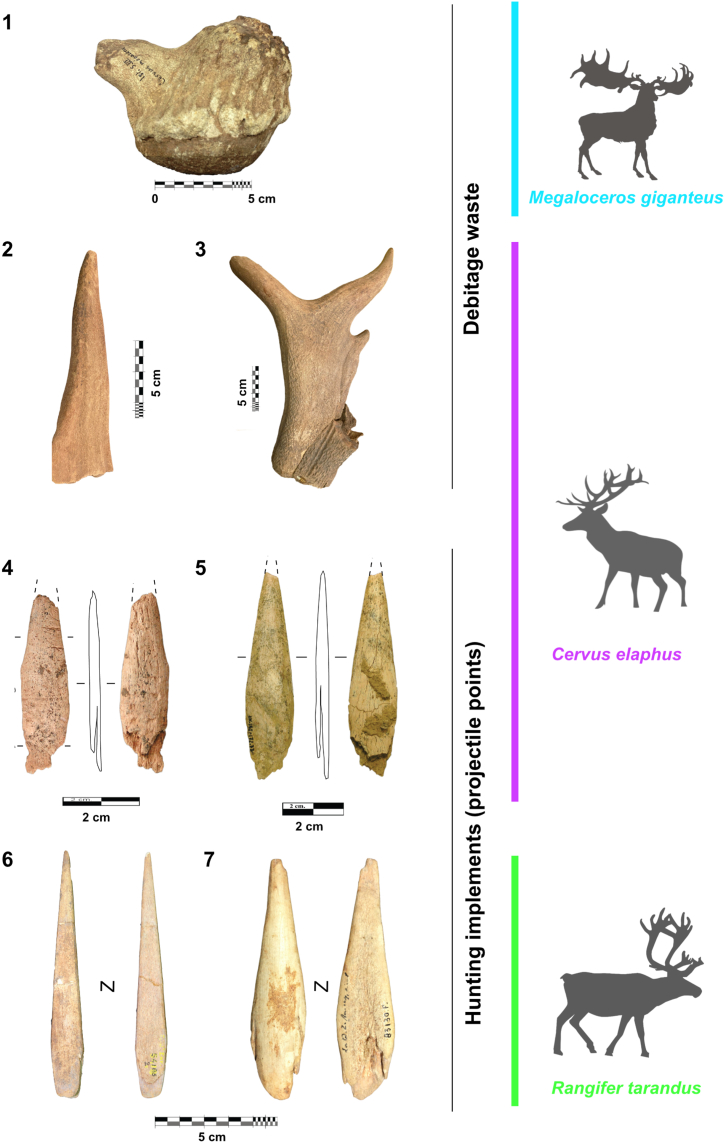
Prehistoric bone implements (debitage waste and projectile points) made on antler from diverse taxa: *Megaloceros giganteus* (1) (Isturitz Cave. France); *Cervus elaphus* (2–5) (2. Satsurblia. 3 Semele Klde. Georgia. 4. Cueva de la Viña. 5. Labeko Koba. Spain); *Rangifer tarandus* (6–7) (6. Abri Poisson. 7. La Quina. France). Items 4 and 5 are not included in this study.

However, antlers can originate from diverse deer species with different ecological requirements and varying behaviour. The exploitation of antlers from at least six taxa in Prehistoric times has been recorded; red deer (*Cervus elaphus*), reindeer (*Rangifertarandus*), giant deer (*Megaloceros giganteus*), Persian fallow deer (*Dama mesopotamica)*, elk (*Alces alces*) and Axis deer (*Axis shansius*) have been documented from a range of UP archaeological sites from Western Europe to Eastern Asia [6,[26], [27], [28], [29]] (Fig. 1). As has been suggested for bone, ivory and teeth objects, identifying the antler's origin at a taxonomic level is therefore critical in improving our knowledge of humans' subsistence, social behaviour, functional, practical and symbolic choices, and the human-animal interface during Prehistoric times [[30], [31], [32], [33], [34], [35], [36], [37]].

Antlers are an exoskeletal appendage characteristic of the Cervidae (deer) family with a yearly cycle of growth, fall and regrowth [[38], [39], [40]]. Shape and size are highly variable between species; thus, their morpho-structural properties are very diverse [[41], [42], [43]]. Such properties undoubtedly restrict their potential technical exploitation. Manufacturing a projectile point requires a fragment of antler that is both long and straight to provide the projectile with symmetry and enough thickness to ensure its solidity and right trajectory. Only developed antlers, specifically the beam parts, of adult individuals from some species can fulfill such requirements [6]. This is likely the reason why some deer documented in several archaeological Pleistocene sites, like European fallow deer (*Dama dama*) and roe deer (*Capreolus capreolus*), never seem to be used for the production of hunting weapons or other tools. Roe deer antlers are generally unsuitable for technical exploitation due to their small size, and the low thickness of compact bone tissue [44]. The European fallow deer, contrary to the Persian subspecies [28], has flat antlers with a less developed beam. Additionally, cervidae taxa occupied various habitats, from open landscapes to closed forests, swamps, and arctic tundra, and from mid to high latitudes, spanning the Eurasian mega continent during the late Pleistocene [[45], [46], [47], [48], [49], [50], [51], [52], [53], [54], [55]]. Thus, both the morpho-structural properties and the ecological distribution of deer limit raw material availability in a given spatio-regional context. Nevertheless, practical constraints don't always explain the choices of Prehistoric societies. We have recent examples of cultural preferences inferred from the selection of one or several taxa [30,[35], [36], [37]]. Selecting certain species and anatomical parts has demonstrated both shared and divergent choices regarding the aesthetic–cum-symbolic set of personal ornaments and decorated bones from Western Europe and the Levant in the Early UP [35,36].

For the majority of antler objects, the designation of raw material is performed by macroscopic analysis [4]. Categorizing osseous tissues' exact taxonomic origin is, however, generally only possible using biomolecular methods, albeit some attempts by X-ray micro-tomography have been made [56] to differentiate between red deer and reindeer antlers. A major difficulty lies in identifying the intensely transformed anatomical blank during the objects’ production, involving the loss of many, if not all, specific diagnostic attributes.

Biomolecular techniques have therefore become invaluable tools to identify the species of raw materials used. Two methods can be employed for this purpose, namely palaeogenomics and palaeoproteomics (through Zooarchaeology by Mass Spectrometry method or “ZooMS”). The latter method uses peptide mass-fingerprinting of collagen to identify the species of osseous fragments. It is widely used in archaeology and palaeontology, with an expanding range of applications. ZooMS was first proposed by Buckley et al. [57] as a method for identifying the species of bone fragments where no morphological indicators are present. It was further developed [58,59] and recently applied for bone tools taxa identification following a non-destructive sampling technique [30]. ZooMS is less invasive and cheaper than aDNA analyses. However, it only allows for discrimination at the family level, and therefore not always accurate enough to identify diverse deer taxa. Conversely, aDNA can provide more accurate data, potentially including the sex and the phylogeny of the exploited species even with little preserved aDNA, something impossible with ZooMS. Ancient DNA can therefore provide unique information about the makers/users of (pre)(historic) bone tools, and even potentially the prey hunted with a single antler projectile.

Nevertheless, despite the importance of the diverse skeletal tissues for prehistoric past societies, palaeogenetics and palaeoproteomics analyses of osseous objects have mainly focused on bone [30,32,33,60] and tooth [34] artefacts. Genetic studies of antlers are mostly restricted to modern specimens in the context of deer conservation (e.g., Refs. [[61], [62], [63], [64], [65]]). Ancient DNA (aDNA) analyses, sometimes in combination with palaeoproteomics, of deer antlers have been restricted to palaeontological sites [65,66]. Analyses of antlers from archaeological contexts have so far been limited to post-Pleistocene periods (Holocene context [67], pre-Viking contexts from Scotland and Scandinavia [68] and Middle Ages [69].

Here, we present the results of the ancient DNA (aDNA) analysis of a set of antler fragments and objects from Palaeolithic and Neolithic archaeological contexts (c. 34-8ka.). These come from a range of sites in South-Western Europe (France, Spain), Central Europe (Austria, Czech Republic), South-Western Asia (Lebanon and Israel), and the Caucasus (Georgia) (Table 1). We obtained aDNA through a minimally invasive sampling method, allowing us to identify the exploited taxa, demonstrating that ancient antler objects can be a reliable long-term source of aDNA. In addition to the antler objects, the method was also applied to a series of bone tools enabling the comparison between antler and cortical bone. The method is combined with a custom-created set of capture baits for the mitochondrial DNA of 52 mammalian species (Supplementary Table 1), based on the most representative taxa of the Eurasian studied regions and the primary sources of human industry. The obtained mitochondrial data have been used to identify the exploited taxa and further explore five individuals’ phylogenies. We quantitatively assess the invasiveness of our new method on the objects by studying their macro-morphology and structure to be able. Macroscopic and microscopic assessments, and as micro-CT scans confirmed that the macro and micro-morphology of objects remains broadly unchanged after sampling, allowing the carrying out of a range of further studies on the objects after sampling, including morphometric, technical, genetic, and radiometric analyses.

**Table 1:** Description of the samples.

ID	Site	Country	layer/Unit	Period	Chronology (available C14 dates)	Raw material	Tool type	Curating institution	References
Dz15136	Dzudzuana	Georgia	Unit D	Early Upper Palaeolithic	34.5–32.2 ka	bone	projectile point	National Georgian Museum	Bar-Yosef et al., 2011
Dz2724	Dzudzuana	Georgia	Unit C Layer 2	Upper Palaeolithic	27.0–24.0 ka	bone	projectile point	National Georgian Museum	Bar-Yosef et al., 2011
Dz19364	Dzudzuana	Georgia	Unit C	Upper Palaeolithic	27.0–24.0 ka	bone	projectile point	National Georgian Museum	Bar-Yosef et al., 2011
Dz19352	Dzudzuana	Georgia	Unit D	Early Upper Palaeolithic	34.5–32.2 ka	antler	projectile point	National Georgian Museum	Bar-Yosef et al., 2011
Dz15129	Dzudzuana	Georgia	Unit C Layer 4	Upper Palaeolithic	27.0–24.0 ka	bone	projectile point	National Georgian Museum	Bar-Yosef et al., 2011
Dz19285	Dzudzuana	Georgia	Unit D	Early Upper Palaeolithic	34.5–32.2 ka	bone	projectile point	National Georgian Museum	Bar-Yosef et al., 2011
ML4529	Mladec	Czech Republic	Aurignacian	Aurignacian	c. 31.0 kyr BP	antler	projectile point	The Anthropos Institut of the Moravian Museum	Teschler-Nicola 2006, Wild et al., 2006
ML4530	Mladec	Czech Republic	Aurignacian	Aurignacian	c. 31.0 kyr BP	ivory	projectile point	The Anthropos Institut of the Moravian Museum	Teschler-Nicola 2006, Wild et al., 2006
ML4533	Mladec	Czech Republic	Aurignacian	Aurignacian	c. 31.0 kyr BP	antler	projectile point	The Anthropos Institut of the Moravian Museum	Teschler-Nicola 2006, Wild et al., 2006
ML4534	Mladec	Czech Republic	Aurignacian	Aurignacian	c. 31.0 kyr BP	antler	projectile point	The Anthropos Institut of the Moravian Museum	Teschler-Nicola 2006, Wild et al., 2006
ML4532	Mladec	Czech Republic	Aurignacian	Aurignacian	c. 31.0 kyr BP	antler	projectile point	The Anthropos Institut of the Moravian Museum	Teschler-Nicola 2006, Wild et al., 2006
Poi1	Abri Poisson	France	Early Aurignacian	Aurignacian	–	antler	projectile point	Musée d'Archéologie National de France	Peyrony 1932
Poi2	Abri Poisson	France	Early Aurignacian	Aurignacian	–	antler	projectile point	Musée d'Archéologie National de France	Peyrony 1932
LQ10	La Quina	France	Early Aurignacian	Early Aurignacian	–	antler	debitage waste	Musée d'Archéologie National de France	L. Henri-Martin 1930, G. Henri-Martin 1956, Dujardin et Kervazo 2010
Fe4	La ferassie	France	Aurignacian	Aurignacian	–	antler	projectile point	Musée d'Archéologie National de France	Peyrony 1932
IST4	Isturitz	France	S-III A⍵	Aurignacian	–	antler	projectile point	Musée d'Archéologie National de France	Normand et Cattelain, 2017
St755	Satsurblia	Georgia	BIV b	Upper Palaeolithic	31.6–32.0 ka	antler	debitage waste	National Georgian Museum	Pinhasi et al., 2014, Tejero et al., 2021
St766	Satsurblia	Georgia	BIV	Upper Palaeolithic	31.6–32.0 ka	antler	debitage waste	National Georgian Museum	Pinhasi et al., 2014, Tejero et al., 2021
St766b	Satsurblia	Georgia	BIII	Upper Palaeolithic	24.3–25.1 ka	antler	debitage waste	National Georgian Museum	Pinhasi et al., 2014, Tejero et al., 2021
St1017	Satsurblia	Georgia	BIV b	Upper Palaeolithic	31.6–32.0 ka	antler	debitage waste	National Georgian Museum	Pinhasi et al., 2014, Tejero et al., 2021
St673_powder	Satsurblia	Georgia	BIII	Upper Palaeolithic	24.3–25.1 ka	antler	debitage waste	National Georgian Museum	Pinhasi et al., 2014, Tejero et al., 2021
St784_powder	Satsurblia	Georgia	BIII	Upper Palaeolithic	24.3–25.1 ka	antler	projectile point	National Georgian Museum	Pinhasi et al., 2014, Tejero et al., 2021
St784	Satsurblia	Georgia	BIII	Upper Palaeolithic	24.3–25.1 ka	antler	projectile point	National Georgian Museum	Pinhasi et al., 2014, Tejero et al., 2021
St694_powder	Satsurblia	Georgia	BIII	Upper Palaeolithic	24.3–25.1 ka	bone	projectile point	National Georgian Museum	Pinhasi et al., 2014, Tejero et al., 2021
St694	Satsurblia	Georgia	BIII	Upper Palaeolithic	24.3–25.1 ka	bone	projectile point	National Georgian Museum	Pinhasi et al., 2014, Tejero et al., 2021
St801	Satsurblia	Georgia	BIII	Upper Palaeolithic	24.3–25.1 ka	bone	projectile point	National Georgian Museum	Pinhasi et al., 2014, Tejero et al., 2021
Dz13771	Dzudzuana	Georgia	Unit C	Upper Palaeolithic	27.0–24.0 ka	bone	projectile point	National Georgian Museum	Bar-Yosef et al., 2011
Dz12076_powder	Dzudzuana	Georgia	Unit C	Upper Palaeolithic	27.0–24.0 ka	bone	projectile point	National Georgian Museum	Bar-Yosef et al., 2011
Dz12076	Dzudzuana	Georgia	Unit C	Upper Palaeolithic	27.0–24.0 ka	bone	projectile point	National Georgian Museum	Bar-Yosef et al., 2011
Dz19307_powder	Dzudzuana	Georgia	Unit C	Upper Palaeolithic	27.0–24.0 ka	antler?	projectile point	National Georgian Museum	Bar-Yosef et al., 2011
Dz19307	Dzudzuana	Georgia	Unit C	Upper Palaeolithic	27.0–24.0 ka	antler?	projectile point	National Georgian Museum	Bar-Yosef et al., 2011
Samele Klde_powder	Samele Klde	Georgia	ind.	Neolithic?	–	antler	debitage waste	National Georgian Museum	–
Samele Klde	Samele Klde	Georgia	ind.	Neolithic?	–	antler	debitage waste	National Georgian Museum	–
CHU1	Chufin	Spain	301	Solutrean	–	antler	harpoon	University of Cantabria, freshly excavated	unpublished
CHU2	Chufin	Spain	305	Solutrean	–	antler	debitage waste	University of Cantabria, freshly excavated	unpublished
CHU3	Chufin	Spain	201	Solutrean	–	antler	projectile point	University of Cantabria, freshly excavated	unpublished
CHU4	Chufin	Spain	304	Solutrean	–	bone	worked bone	University of Salamanca, freshly excavated	unpublished
KS3; RGM.1333607	Ksar Akil	Lebanon	V	Epipalaolithique	26.210 + 130-120: 30.000 cal BP	bone	awl	Naturalis Biodiversity Center, NL	Ewing 1948, Newcomer 1974, Bosch et al., 2015
KS6; RGM.1333610	Ksâr ‘Akil	Lebanon	XXVII	cf. Levantine Mousterian	40.550 + 350/−310 cal BP	bone	awl	Naturalis Biodiversity Center, NL	Ewing 1948, Newcomer 1974, Bosch et al., 2015
NR1	Nahal Rahaf	Israel	layer 5	Arkov-Divshon	31.462 ± 230 Ka	bone	awl	Israel Antiquities Authority	Shemer et al., 2023
NR2	Nahal Rahaf	Israel	layer 7b	Arkov-Divshon	31.810 ± 110 ka	bone	awl	Israel Antiquities Authority	Shemer et al., 2023
StEx1	Satsurblia	Georgia	AIIb	Upper Palaeolithic	17.2–17.9 ka	bone	awl	National Georgian Museum	Pinhasi et al., 2014, Tejero et al., 2021
StEx2	Satsurblia	Georgia	AIIb	Upper Palaeolithic	17.2–17.9 ka	bone	awl	National Georgian Museum	Pinhasi et al., 2014, Tejero et al., 2021
StEx3	Satsurblia	Georgia	AIIb	Upper Palaeolithic	17.2–17.9 ka	bone	projectile point	National Georgian Museum	Pinhasi et al., 2014, Tejero et al., 2021
TB1	Tito Bustillo	Spain	UE103	Magdalenian	14.890 + 410 BP	antler	projectile point	University of Salamanca, freshly excavated	Alvarez-Fernández et al., 2022
TB2	Tito Bustillo	Spain	UE104	Magdalenian	14.890 + 410 BP	antler	projectile point	University of Salamanca, freshly excavated	Alvarez-Fernández et al., 2022
TB3	Tito Bustillo	Spain	UE105	Magdalenian	14.890 + 410 BP	antler	projectile point	University of Salamanca, freshly excavated	Alvarez-Fernández et al., 2022
GI-5866	La Garma	Spain	Lower Galery (I)	Magdalenian	14.050 ± 110	antler	projectile point	University of Cantabria, freshly excavated	Arias and Ontañón 2012, 2014
GI-5817	La Garma	Spain	Lower Galery (III)	Magdalenian	13.810 ± 160	antler	projectile point	University of Cantabria, freshly excavated	Arias and Ontañón 2012, 2014
GI-7963	La Garma	Spain	Lower Galery (I)	Magdalenian	14.050 ± 110	bone	faunal remain	University of Cantabria, freshly excavated	Arias and Ontañón 2012, 2014
GI-5835	La Garma	Spain	Lower Galery (I)	Magdalenian	14.050 ± 110	antler	projectile point	University of Cantabria, freshly excavated	Arias and Ontañón 2012, 2014
GI-7964	La Garma	Spain	Lower Galery (I)	Magdalenian	14.050 ± 110	bone	faunal remain	University of Cantabria, freshly excavated	Arias and Ontañón 2012, 2014
GI-5869	La Garma	Spain	Lower Galery (I)	Magdalenian	14.050 ± 110	bone	faunal remain	University of Cantabria, freshly excavated	Arias and Ontañón 2012, 2014
GI-7968	La Garma	Spain	Lower Galery (III)	Magdalenian	13.810 ± 160	antler	projectile point	University of Cantabria, freshly excavated	Arias and Ontañón 2012, 2014
GI-7969	La Garma	Spain	Lower Galery (III)	Magdalenian	13.810 ± 160	antler	projectile point	University of Cantabria, freshly excavated	Arias and Ontañón 2012, 2014

## 2 Results

We captured mitochondrial DNA from 50 bone and antler items. For 34 of those (72 %), we were not able to identify any non-human mammalian mitochondrial DNA. For seven of those samples (14 %), the species identified contradicted the preliminary visual analysis, which suggested that the items were made of antler, but the genetically identified species did not possess such exoskeletal appendages (*Sus scrofa*, *Bos taurus*, and *Capra hircus*). These results can be explained by the conservation of the items in the museum. These three species are consistent with those used to make animal-based glues, commonly used in museum conservation [70]. Finally, for 17 of the items (34 %), it was possible to confidently identify the source species. While most of these yielded a very low mitochondrial coverage (<6x), 7 yielded more data (5 of which were made of antler), enabling further phylogenetic analyses. We checked the deamination values of the human DNA by selecting the human-aligned reads using samtools and checking the deamination values using mapdamage 2.2.1 [71]. In any case, these values were greater than 0.01, suggesting the absence of substantial ancient human DNA in the samples.

**Fig. 2:**
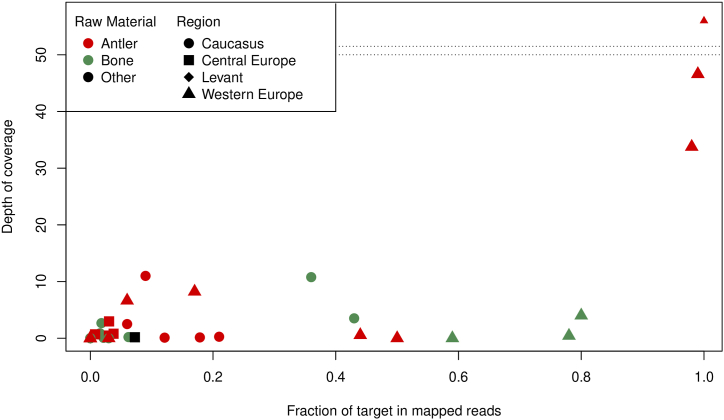
Scatter plot representing the samples included in the analyses. Y axis represents the target's coverage depth, and the X axis represents the fraction of aligned reads of each sample corresponding to the target. Samples from bone tend to have higher fractions of recovered reads; however, the samples with the highest depth of coverage are all from antlers.

Out of the 28 antler items tested, 8 (29 %) gave enough results for a positive taxon identification. Bone samples were successful in 10 out of 20 (50 %). This confirms that antler is indeed a reliable source of aDNA, albeit not as efficient at preservation as bone (Fig. 2).

For the five samples for which extracts were obtained using both the traditional powdering method and the minimally-invasive method (Table 3), species identification was possible in two cases. Although the powdering method yielded a higher number of reads and a consequently higher coverage, the ability to identify species seems to be similar with both methods. It may therefore be recommended to use the powdering method in borderline samples, but the minimally invasive method seems to perform well enough when aDNA is fairly well preserved.

## 3 Discussion

Osseous tools are a fundamental proxy for understanding the subsistence and cultural networks of Palaeolithic peoples [31,72]. The correct determination of the species origin is fundamental to gaining insights into the origin of the raw material employed, especially in areas where no or very few exploited animals are present [6,56]. Recently, proteomics, especially ZooMS, has emerged as a reliable method to identify the taxon of animal-made artefacts with very little input material required [30]. Despite huge recent improvements, ZooMS only enables the identification of taxa, without enabling further phylogenetic inference, and may lack resolution at the species level. Consequently, ancient DNA appears as a reliable tool to address questions regarding the exploitation of bone-tools in prehistory, when the key questions relate to species-origin and the possible existence of genetic similarities relating to trading networks. Previous work on personal ornaments has demonstrated that it is feasible to obtain the DNA of the wearers/makers of the ornaments [34]. It is therefore also envisageable to recover the DNA and identify the preys hunted with osseous weapons, something which, to this day, remains subject to speculation.

It is well-accepted that ancient DNA preservation is related to climatic conditions, and that recovering DNA from warm and humid climates is particularly challenging [73]. Here we observe that none of the four here-studied artefacts from the Middle East (namely those from Nahal Rahaf and Ksâr ‘Akil) have yielded DNA-based taxonomic identification. In contrast, for 8 out of 15 from the Cantabrian region, this was possible. Therefore, it is clear that the success of the presented project is strongly determined by the environmental conditions determining the preservation of aDNA. In this study, however, we bring a new insight into sample efficiency related to the storing conditions and manipulation. Focusing on the temperate region samples, we observe that while 9 out of 31 pieces stored in collections yielded results. More importantly, the samples kept in museums for over 100 years and handled abundantly failed systematically (this includes all samples from the French collection). The items from Mladec were also stored and handled for a long time period prior to analyses. These seem to have been treated with animal-based glues at some point in their conservation history, as reflected in the taxa identified through the aDNA analysis. In stark contrast, the recently excavated samples from Tito Bustillo and Chufín (both caves in a temperate region) yielded excellent results, thereby confirming the suitability of pre-historic antler implements as a source of aDNA.

The unique status of Cervidae in Late Pleistocene hunter-gatherer societies is further reflected by the finding of many personal ornaments made from perforated red deer teeth [74,75], and through the frequent representations of red deer in Southeast European parietal and portable art [[76], [77], [78]], as well as that of other cervid species [79,80]. Due to the diversity of the various cervid species’ antlers' availability and technical constraints, there is high value in identifying the selected taxa in each region, site and layer, thereby allowing us to differentiate ecological and technological choices against the cultural selections of our Prehistoric ancestors. Nevertheless, we can only build an objective database solid enough on exploited deer taxa for technical purposes by applying aDNA analyses. Here we have successfully recovered phylogenetic information from 5 Iberian implements plausible with the use of local *Cervus elaphus*.

Although biomolecular analyses are of great value, recent studies stress the significance of evaluating potential effects of various sampling methods on bone tools [32,81,82]. Our study demonstrates that the minimally-invasive aDNA method implemented by Harney et al. [83] for human teeth (itself a modification of [84]) can be adapted and applied to bone and antler tools, when sampling by powdering is not possible. Another minimally-invasive method has recently been presented by Essel et al. [34]. The team has successfully extracted aDNA of the raw material animal as well as that of the users/makers of the object, with no significant observable morphological modifications to the object itself. However, it must be noted that the time and equipment required to perform such extractions make it extremely challenging to perform in any environment outside the laboratory. In contrast, using our here-presented method enabled us to perform most of the extractions directly at the storage location of the item or even at the site itself, thereby not requiring the transportation or export of any items which can be necessary in some cases, especially when studying rare items that may not be possible to remove from collections.

The oldest antlers to yield aDNA so far came from Palaeontological contexts of around 12ka [65]. Our study extends this range significantly, setting the stage to improve our knowledge of Upper Palaeolithic societies from the earlier *H. sapiens* groups permanently settling in Eurasia (*C*. 45,000 years ago) to recent Prehistoric times. Our results demonstrate that pre-Holocene antler implements can be a source of aDNA. While bone and teeth have, thus far, been the primary tissues used to obtain aDNA, we hereby confirm worked antler as another potential source. Given the importance of antlers as a raw material for the hunter-gatherer groups at the end of the Pleistocene, but also for later societies up until the Middle Ages, it is critical to obtain from them as much data as possible by combining archaeological and biomolecular methods.

## 4 Material and methods

The analysed assemblage comprises 50 Upper Palaeolithic items encompassing hunting implements (projectile points and one harpoon), blanks, production wastes and domestic tools (awls) (Table 1). All items were studied with the full permission of the respective curators and collection caretakers.

### 4.1 Ancient DNA

DNA sampling was performed using two methods. Around ∼50 mg of powder from the object's interior for some pieces were collected by drilling. The DNA was then extracted from powder following the protocol outlined by Ref. [85] with modifications described in Ref. [86], namely the replacement of the Qiagen Minelute column custom constructions for DNA purification with columns from the Roche High Pure Viral Nucleic Acid kit. Most items were sampled using the minimally-destructive extraction procedure presented here. It is based on the protocol described by Ref. [83] with several modifications detailed below.

The extractions were performed at the location of sample storage and inside the cave in the case of La Garma (Spain). The environment in which it was served was cleaned as thoroughly as possible: surfaces were wiped with a dilute (about 1.2 %) bleach solution and covered with a bleach-cleaned aluminium foil. We verified that no PCR was ever performed in the same space to avoid potential contamination.

The first step consisted of cleaning each object by wiping with a bleach solution (about 1.2 %) and then rinsing thoroughly with absolute ethanol. The pieces were then exposed to short-wave (254 nm) UV light for 10 min on each surface.

Unlike the procedure described in the Harney et al. protocol [83], the samples were not wrapped in Parafilm, but entirely submerged in extraction buffer. The exception was the samples stored at the *Musée d’Archéologie Nationale* (France), where the pieces were wholly wrapped in parafilm except for leaving a small window exposed (∼2–4 cm^2^). The smallest possible container was selected to fit the whole piece comfortably with as little spare space as possible. The possible containers were 5 ml, 15 ml and 50 ml Eppendorf DNA LoBind tubes and sterile plastic bags.

In some cases, the object was submerged for 20 min in extraction buffer, for a pre-digestion. The initial lysate was then discarded to remove the potential external DNA contamination. This was only performed for the later batch of samples containing the items from Chufín, Tito Bustillo-Área de Estancia, Nahal Rahaf 2 and Satsurblia (experimental items). The items were then re-submerged in an extraction buffer, the volume of which was adapted for each piece. The minimum amount that enabled the pieces to be fully submerged ranged between 1.0 and 15.0 ml. The extraction was performed in room-temperature to warm conditions at ∼35 degrees C, with the liquid in the tubes moved around gently at regular 15-min intervals, while monitoring the effect of the buffer on the piece's surface condition. The duration of the extraction was adapted for each item. In all cases it was stopped at the latest as soon as any effect of digestion on the piece became visible or evidence of significant dissolution was detected through a marked change in the colour of the extraction buffer, as it is unfortunately not possible to objectively measure the lack of damage. Individual digestion times are given in Table 2, ranging from 0.5 to 2.5 h. The resulting lysate was then stored in a freezer.

**Table 2:** Sequencing results.

ID	Pre-digestion	Digestion time	Sequenced reads	Human aligned and filtered	Human depth (x)	Human Damage (3')	Animal aligned and filtered	Animal depth (x)	Damage animal (3')	Human:animal reads	Proportion of target animal reads in run	Species assessment	Overall assessment
**Dz15136**	2.5	NO	7596370	30117	144.77	0	556	2.67	0.46	54.17	0.00007	*Bos taurus*	Possible species ID
**Dz2724**	2.5	NO	3985647	955	3.93	0.02	21	0.07	0.02	45.48	0.00001	*Bos taurus*	Fail
**Dz19364**	2.5	NO	3486590	904	4.00	0	60	0.20	0.02	15.07	0.00002	*Cervus elaphus*	Possible species ID
**Dz19352**	2.5	NO	4894516	1190	5.00	0	164	0.1	0.08	7.26	0.00003	*Alces alces*	Possible species ID
**Dz15129**	2.5	NO	66595	1	–	0.01	–	–	0.01	–	–	*-*	Fail
**Dz19285**	2.5	NO	3440798	10240	46.73	0.01	164	0.87	0.01	62.44	0.00005	*Sus scrofa*	Fail: Implausible species ID; must be animal glue contaminant
**ML4529**	2.5	NO	3594736	4592	18.14	0.02	181	0.8	0.02	25.37	0.00005	*Sus scrofa*	Fail: Implausible species ID; must be animal glue contaminant
**ML4530**	2.5	NO	5974246	741	2.76	0.01	58	0.17	0.02	12.78	0.00001	*Bos taurus*	Fail: Implausible species ID; must be animal glue contaminant
**ML4533**	2.5	NO	5223363	4454	16.62	0.02	139	0.47	0.03	32.04	0.00003	*Bos taurus*	Fail: Implausible species ID; must be animal glue contaminant
**ML4534**	2.5	NO	4144995	28306	134.02	0.03	892	2.97	0.03	31.73	0.00022	*Bos taurus*	Fail: Implausible species ID; must be animal glue contaminant
**ML4532**	2.5	NO	3737677	22929	94.54	0.01	181	0.69	0.01	126.68	0.00005	*Capra hircus*	Fail: Implausible species ID; must be animal glue contaminant
**Poi1**	2.0	NO	5406771	135	1.70	0	–	–	0.01	–	–	*-*	Fail
**Poi2**	2.0	NO	8950423	164	0.63	0.03	–	–	0.00	–	–	*-*	Fail
**LQ10**	2.0	NO	4177141	135	0.57	0.03	–	–	0.00	–	–	*-*	Fail
**Fe4**	2.0	NO	6870813	1843	8.55	0.01	–	–	0.01	–	–	*-*	Fail
**IST4**	2.0	NO	1269927	–	–	0	–	–	0.10	–	–	*-*	Fail
**St755**	2.5	NO	13668029	532	2.43	0.04	–	–	0.00	–	–	*-*	Fail
**St766**	2.5	NO	5702910	112	0.49	0	–	–	0.00	–	–	*-*	Fail
**St766b**	2.5	NO	4071809	138	0.69	0.03	30	0.14	0.09	4.60	0.00001	*Bos taurus*	Fail: Implausible species ID; must be animal glue contaminant
**St1017**	2.5	NO	4699441	77	0.35	0	–	–	0.20	–	–	*-*	Fail
**St673_powder**	18.0	NO	8476281	126	1.00	0.01	1501	5.4	0.40	0.08	0.00018	*Cervus elaphus*	Possible species ID
**St784**	2.5	NO	8019576	877	3.50	0.01	–	–	–	–	–	*-*	Fail
**St694**	2.5	NO	13780889	12443	55.3	0	779	2.5	0.35	15.97	0.00006	*Bison bonasus*	Possible species ID
**St801**	2.5	NO	10825776	30482	172.00	0.01	2976	11	0.44	10.24	0.00027	*Capra hircus*	Possible species ID
**Dz13771**	2.5	NO	6965180	2441	10.70	0.01	1398	10.78	0.42	1.75	0.00020	*Capra hircus*	Possible species ID
**Dz12076**	2.5	NO	7460673	13767	65.4	0.02	–	–	–	–	–	*-*	Fail
**Dz19307**	2.5	NO	7050983	9560	42.00	0.01	–	–	–	–	–	*-*	Fail
**Samele Klde**	2.5	NO	8555243	313	0.97	0	83	0.26	–	3.77	0.00001	*Cervus elaphus*	Possible species ID
**CHU1**	1.5	YES	4444816	4204	0.69	0.01	3	–	0.12	1401.33	0.00000	*-*	Fail
**CHU2**	1.5	YES	4340206	10742	0.75	0.01	2200	8.222	0.39	4.88	0.00051	*Cervus elaphus*	Possible species ID
**CHU3**	1.5	YES	5786327	30464	1.11	0.02	1876	6.65	0.25	16.24	0.00032	*Cervus elaphus*	Possible species ID
**CHU4**	1.5	YES	1367638	2497	0.49	0.04	29371	176.91	0.34	0.09	0.02148	*Cervus elaphus*	Possible species ID
**KS3; RGM.1333607**	2.0	YES	4854073	255	14.75	0	0	–	–	–	–	*-*	Fail
**KS6; RGM.1333610**	2.0	YES	4274500	2278	6.37	0.02	1	–	0.01	2278.00	0.00000	*-*	Fail
**NR1**	2.0	YES	5041283	1262	5.94	0.01	0	–	0.01	–	–	*-*	Fail
**NR2**	2.0	YES	4737747	3439	2.19	0	0	–	0.01	–	–	*-*	Fail
**StEx1**	2.0	YES	5737159	14018	–	0	33	–	0.18	424.79	0.00001	*-*	Fail
**StEx2**	2.0	YES	4605353	557	–	0.02	17	–	0.23	32.76	0.00000	*-*	Fail
**StEx3**	2.0	YES	5015779	1504	12.33	0	1138	3.51	0.33	1.32	0.00023	*Bison bonasus*	Possible species ID
**TB1**	1.5	YES	157358	184	0.00	0.11	147	0.56	0.43	1.25	0.00093	*Cervus elaphus*	Possible species ID
**TB2**	1.5	YES	33254	72	0.16	0	13904	46.60	0.36	0.01	0.41812	*Cervus elaphus*	Possible species ID
**TB3**	1.5	YES	3886632	196	1.86	0.01	9708	33.77	0.43	0.02	0.00250	*Cervus elaphus*	Possible species ID
**GI-5866**	2.0	NO	5506646.00	773.00	3.40	0.04	–	–	0.51	–	–	–	Fail
**GI-5817**	2.0	NO	8376119.00	3529.00	15.34	0	–	–	0.01	–	–	–	Fail
**GI-7963**	2.0	NO	8015545.00	335.00	1.12	0.01	1349	4.01	0.25	0.25	0.00	Cervus elaphus	Possible species ID
**GI-5835**	2.0	NO	8290699.00	18773.00	95.23	0.01	–	–	0.01	–	–	–	Fail
**GI-7964**	2.0	NO	2778282.00	63.00	0.12	0.05	230	0.43	0.23	0.27	0.00	Cervus elaphus	Possible species ID
**GI-5869**	2.0	NO	790674.00	11.00	–	0	16	–	0.00	0.69	0.00	–	Fail
**GI-7968**	2.0	NO	6211225.00	492.00	2.20	0.02	13	–	0.08	37.85	0.00	–	Fail
**GI-7969**	2.0	NO	3721428.00	13.00	–	0	13	–	0.00	1.00	0.00	–	Fail
**EXTRACTION-BLANK1**	–	–	196250	40	–	0	–	–	0.40	–	–	*-*	Blank
**LIBRARY-BLANK1**	–	–	66595	1	–	0	–	–	–	–	–	*-*	Blank
**EXTRACTION-BLANK2**	–	–	285807	39	–	0	–	–	–	–	–	*-*	Blank
**LIBRARY-BLANK2**	–	–	278834	2	–	0	–	–	–	–	–	*-*	Blank
**EXTRACTION-BLANK3**	–	–	941044	18	–	0	–	–	–	–	–	*-*	Blank
**LIBRARY-BLANK3**	–	–	620282	8	–	0	–	–	–	–	–	*-*	Blank
**EXTRACTION-BLANK4**	–	–	578274	45	0.23	0	–	–	–	–	–	*-*	Blank
**EXTRACTION-BLANK5**	–	–	52983	5	–	0	–	–	–	–	–	*-*	Blank
**LIBRARY-BLANK4**	–	–	307915.00	2.00	–	0	–	–	–	–	–	–	Blank
**EXTRACTION-BLANK6**	–	–	3238.00	69.00	–	0	69	–	0.00	1.00	0.02	–	Blank
**LIBRARY-BLANK5**	–	–	11907	9	–	0	9	–	0	1.00	0.00076	*-*	Blank

**Table 3:** Comparison of minimally-invasive method with the traditional drilling.

ID	Extraction method	Sequenced reads	Human aligned and filtered	Human depth (x)	Animal aligned and filtered	Animal depth (x)	Damage (3')	Human:animal reads	Species assessment	Overall assessment
St784	Powder	4628525	134	0.48	–	–	–	–	*-*	Fail
	Minimally invasive	8019576	877	3.50	–	–	–	–	*-*	Fail
St694	Powder	8210125	300	1.15	1534	5.60	0.35	0.1956	*B. bonasus*	Positive species ID
	Minimally invasive	13780889	12443	55.3	779	2.50	0.35	15.9730	*B. bonasus*	Positive species ID
Dz12076	Powder	9203358	1480	6.20	–	–	–	–	*-*	Fail
	Minimally invasive	7460673	13767	65.4	–	–	–	–	*-*	Fail
Dz19307	Powder	10220258	582	2.50	–	–	–	–	*-*	Fail
	Minimally invasive	7050983	9560	42.00	–	–	–	–	*-*	Fail
Samele Klde	Powder	10147591	9595	45.8	1332	5.10	0.17	7.2035	*C. elaphus*	Positive species ID
	Minimally invasive	8555243	313	0.97	83	0.26	–	3.7711	*C. elaphus*	Positive species ID

These lysates were then brought to the ancient DNA laboratory at the University of Vienna, and further processed in a dedicated ancient DNA clean room. The lysate clean-up was performed following Dabney et al. (2013) [85] with the modifications described in Harney et al. (2021) [87]. As most samples resulted in more than 1 ml of extraction buffer, a ratio of 13:1 was used to calculate the amount of binding buffer required for optimal binding of the DNA to the silica columns, and the entire mixture ran through the same column.

Subsequently, double-stranded libraries were built from 25.0 μl of extract, according to Meyer and Kircher [88]. Qiagen MinElute PCR Purification kits were used for the intermediate clean-up steps. The libraries were double-indexed and amplified with the NebNext Q5U Master Mix DNA Polymerase (NEB) using a number of cycles calculated employing the qPCR analysis of 1 μl of the library. Indexed libraries were captured using a custom built capture kit for the mitochondrial DNA of 52 mammalian species (Supplementary Table 1). This capture kit has been designed by the team in Vienna and produced by myBaits (Arbor Biosciences) (table SI1). This capture kit allows screening for an extended list of species simultaneously, extending the possibilities to recover aDNA and improving the discrimination capabilities, allowing species-specific hits and better discriminating between species from the same family. This was then shallow-sequenced as part of a larger pool of samples on a single lane of a NovaSeq SP system.

### 4.2 Bioinformatics

Sequenced reads were processed after demultiplexing. Sequenced adapters and short reads below 30 were discarded using Cutadapt 4.2 [89]. The remaining reads were aligned against 40 representative mammalian species in a competitive mapping (list) with bwa aln 0.7.17 [90], disabling seeding and with a gap penalty open of 2. The aligned reads were filtered by quality with samtools 1.16.1 [91], setting minimum mapping quality of 30 and removing duplicates with Picard-tools 2.27.5 [92]. The remaining reads were inspected with mapdamage 2–2.2.1 [71] to determine the deamination patterns and with qualimap 2.2.1 [93] to inspect the results of the competitive mapping. Non-human species were considered positively identified when more than 50 reads could be mapped to the genome of a particular species. When more than one hit was present per sample, we focused on the dominant taxon (the one with the most mapped reads). We therefore considered this as the source. To confirm each of the assignations we examined all the aligned reads with BLAST 2.14.1 [94] using the whole NCBI nt dataset, the assigned hits were examined with the LCA algorithm from MEGAN 6.23.3 [95] to confirm the assignations and discard cross-mappings.

Only samples which yielded more than 500 mammalian aDNA reads were further analysed. For these, we generated a consensus sequence with ANGSD [96]. The consensus sequences were aligned with other present-day and modern animal sequences with Clustal Omega 1.2.4 [97], as performed in multiple projects assessing the mtDNA diversity of Pleistocene fauna [[98], [99], [100], [101]] and we then performed a Maximum likelihood (ML) tree with the alignment using MEGA 10.2.4 [102] with partial deletion and 100 bootstrap replications, 95 % partial deletion and GTR substitution model. All trees were plotted with MEGA.

## Funding sources

Research of J.-M. T. is supported by a project of the Meitner Program of the Austrian Science Fund (10.13039/501100002428FWF) (Project: *Osseous Hunting Weapons of Early Modern Humans in Eurasia.* Number M3112) and the program Ramón y Cajal of the Spanish MCIN/10.13039/501100011033AEI (MCIN/AEI/10.13039/501100011033. Project Number RYC2021-033759-I) and the European Community (NextGenerationEU»/PRTR). The 10.13039/501100003065University of Vienna Research Platform: Mineralogical Preservation of the Human Biome from the Depth of Time (MINERVA) supported the whole project. J.-M. T., P. G., and O. C. benefited from a Seed Grant from the HEAS (Human Evolution and Archaeological Sciences) of the 10.13039/501100003065University of Vienna (Project: *Assessing the differential DNA preservation in Palaeolithic sediments and osseous tools from museum collections*). D. M. B. supported by a Seal of Excellence Fellowship of the 10.13039/501100001822Austrian Academy of Sciences (‘TechnoBeads’ project no. 101061287). P. R. N. benefited from funding from the 10.13039/501100003065University of Vienna and the Land Niederösterreich, Abteilung Wissenschaft & Forschung (project K3–F-530/005–2021). Research at La Garma (P.A. and R.O) is included in the R&D project PID2020-112832RB-I00, funded by the 10.13039/501100004837Spanish Ministry of Science and Innovation (MCIN/AEI/10.13039/501100011033). Research at Tito Bustillo Cave (E.A.F, M.C. and J.T.) is included in the Project PID2020-114462GB-I00/AEI/10.13039/ 501100011033, funded by the Spanish Ministry of Science and Innovation.

## Data availability statement

All sequenced genetic data is available at ENA through the accession number PRJEB61082.

## CRediT authorship contribution statement

**José-Miguel Tejero:** Writing – review & editing, Writing – original draft, Visualization, Validation, Supervision, Resources, Project administration, Methodology, Investigation, Funding acquisition, Formal analysis, Data curation, Conceptualization. **Olivia Cheronet:** Writing – review & editing, Writing – original draft, Visualization, Validation, Supervision, Resources, Methodology, Investigation, Funding acquisition, Formal analysis, Data curation, Conceptualization. **Pere Gelabert:** Writing – review & editing, Writing – original draft, Visualization, Validation, Supervision, Resources, Methodology, Investigation, Funding acquisition, Formal analysis, Data curation, Conceptualization. **Brina Zagorc:** Writing – review & editing, Investigation. **Esteban Alvarez:** Writing – review & editing, Investigation. **Pablo Arias:** Writing – review & editing, Investigation, Funding acquisition. **Aline Averbouh:** Writing – review & editing, Investigation. **Guy Bar-Oz:** Writing – review & editing, Investigation. **Omry Barzilai:** Writing – review & editing, Investigation. **Anna Belfer-Cohen:** Writing – review & editing, Investigation. **Marjolein D. Bosch:** Writing – review & editing, Investigation, Funding acquisition. **Florian Brück:** Writing – review & editing, Investigation, Formal analysis. **Marián Cueto:** Writing – review & editing, Investigation. **Martin Dockner:** Writing – review & editing, Investigation, Formal analysis. **Josep Maria Fullola:** Writing – review & editing, Investigation. **Diego Gárate:** Writing – review & editing, Investigation. **Michael Giannakoulis:** Writing – review & editing, Formal analysis. **Cynthia González:** Writing – review & editing, Investigation. **Nino Jakeli:** Writing – review & editing, Investigation. **Xavier Mangado:** Writing – review & editing, Investigation. **Tengiz Meshveliani:** Writing – review & editing, Investigation. **Petr Neruda:** Writing – review & editing, Investigation. **Philip Nigst:** Writing – review & editing, Investigation, Funding acquisition. **Roberto Ontañón:** Writing – review & editing, Investigation, Funding acquisition. **Maayan Shemer:** Writing – review & editing, Investigation. **Petra G. Šimková:** Writing – review & editing, Investigation, Formal analysis. **Jesús Tapia:** Writing – review & editing, Investigation. **Marta Sánchez de la Torre:** Writing – review & editing, Investigation. **Catherine Schwab:** Writing – review & editing, Investigation. **Gerhard Weber:** Writing – review & editing, Investigation, Formal analysis. **Ron Pinhasi:** Writing – review & editing, Writing – original draft, Supervision, Methodology, Investigation, Funding acquisition, Conceptualization.

## Declaration of competing interest

The authors declare that they have no known competing financial interests or personal relationships that could have appeared to influence the work reported in this paper.
