# Supplementary material for: Cervidae antlers exploited to manufacture prehistoric tools and hunting implements as a reliable source of ancient DNA
Source: Heliyon. 2024 May 24;10(11):e31858. doi: 10.1016/j.heliyon.2024.e31858 (PMC11154607; doi:10.1016/j.heliyon.2024.e31858)
Supplement: Multimedia component 1 [file mmc1.docx]

**Supporting Information**

**Macro and micro morphological analysis of objects**

A comprehensive technical analysis of some of the pieces studied (Isturitz, La Quina, Abri Poisson, Tito Bustillo, Satsurblia, Dzudzuana, Nahal Rahaf 2, and Ksâr ‘Akil) was performed before and after the DNA extraction independently. Technical analysis is based on the assessment of the operational sequence and follows several distinct steps [[1,2]](https://paperpile.com/c/i20Pps/haFbY+zYjXt), including the identification of manufacture and use wear marks.

From a visual macro analysis, we could document the identical marks before and after the extraction (fig. SI2), thus allowing us to reach the same conclusion regarding the technological considerations (reconstruction of the operational sequence of manufacture and use, by functional macro-fractures, of the items).

Moreover, to identify the magnitude of damage caused by the extraction process, the items were scanned at the Vienna µCT Lab before and after extraction using an industrial Viscom X8060 NDT scanner (scanning parameters: 110-140 kV, 280-410 mA, 1400-2000 ms, 0.75 mm copper filter with a voxel size 23 µm). To obtain 3D surface models of the item, virtual segmentation of the µCT data was performed with Amira software ([www.thermofisher.com](http://www.thermofisher.com/)). To further determine possible changes caused by our extraction method, we analysed the surfaces in Geomagic Design X 64 ([www.3dsystems.com](http://www.3dsystems.com/)). 3D models were aligned according to homologous landmark sets on sufficiently visible morphological structures of both surfaces, and differences were assessed.

Using µCT imaging, we assessed the impact of the minimally invasive extraction on the integrity of the items. According to further 3D analyses of one of the items, scanned at a resolution of 23 µm, the average erosion of the surface is between 0 and 200 µm (the large yellow areas in fig. SI1). Only in very limited areas, the maximum surface erosion caused by our approach reaches 300 µm. The few red spots visible in figure SI1 represent areas with material loss of 1 mm and beyond, thus areas where the extraction process caused sediment removal (as visually assessed). In addition, 14 of the items were closely examined with a 3D microscope before and after extraction. Technologically critical marks remained visible (fig. SI3).

**Figure SI1.** Micro-CT scan of StEx1 after sampling. Surfaces coloured in green are unmodified. The predominant yellow to light orange colours show a moderate surface modification between 0.100-0.300 mm depth, resulting from adhered sediment removal.


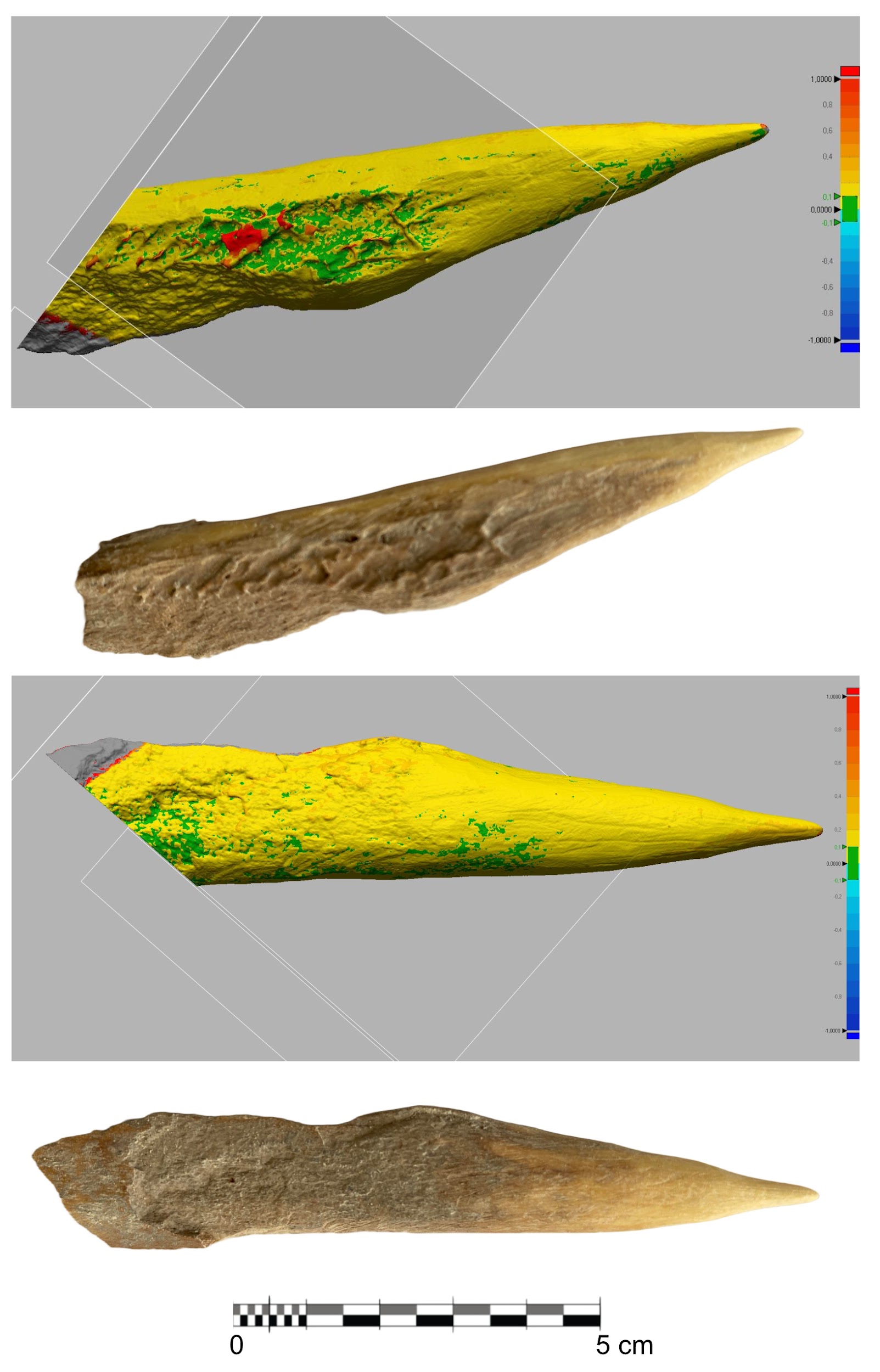


**Figure SI2.** Hunting implements (projectile points) from Satsurblia (Georgia) before and after sampling.
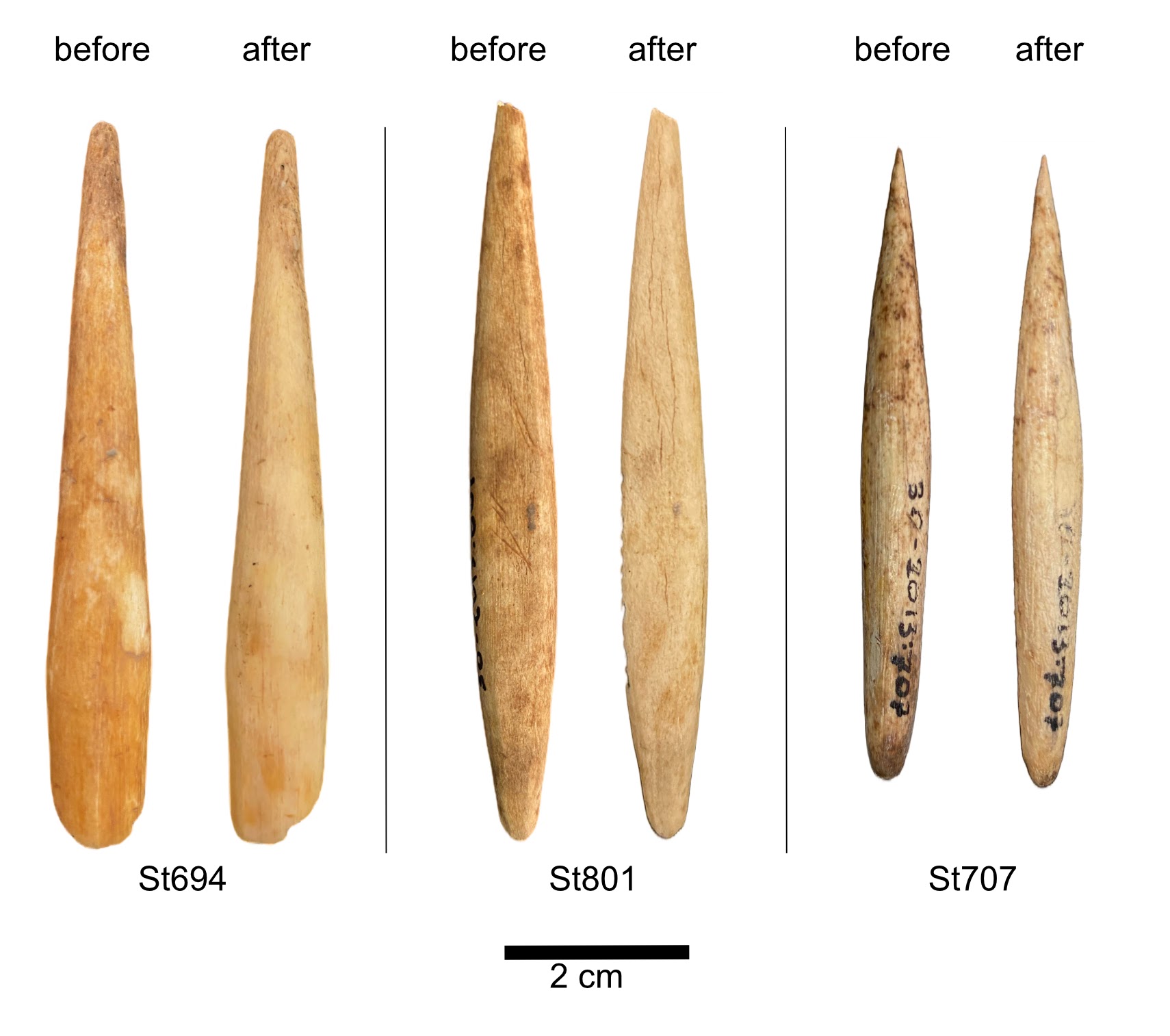


**Fig. SI3.** Hunting implements (projectile points) from Cueva Chufín, Tito Bustillo (Spain), Ksâr ‘Akil (Lebanon), and Nahal Rahaf 2 (Israel), a before and after sampling.

**
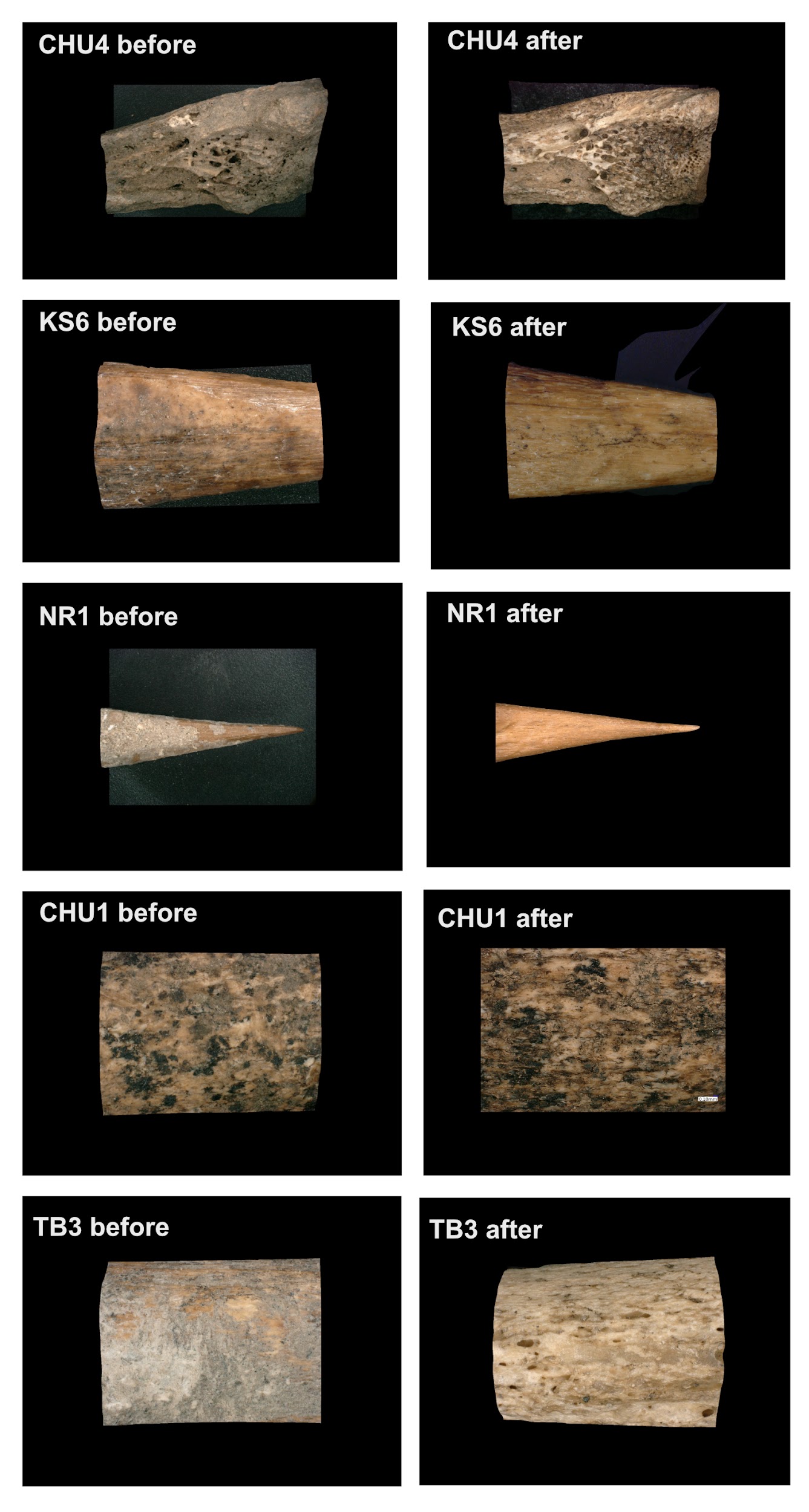
**

**Phylogenetic analyses**

We constructed consensus sequences of the *Cervus elaphus* mitochondrial genome using ANGSD calling the majority allele [[3]](https://paperpile.com/c/i20Pps/tbkRV) and using the sequence NC_007704.2 as the reference genome. The consensus sequences (CHU2, CHU3, CHU4, TB3 and TB2) were aligned with other modern and ancient Cervidae mtDNA [[4-10]](https://paperpile.com/c/i20Pps/VFwHW+Q7VrE+N1E2M+6Z4u4+HMriB+gSSZs+QmRdW), and the phylogenetic relationships were examined with a Maximum Likelihood (ML) tree using 100 boostrap replicates, a 95% partial deletion and GTR substitution model (fig. SI4). We observe that the Iberian samples from Chufín and Tito Bustillo are placed within the Western Clade A of *Cervus elaphus* (fig. SI4) and cluster with other samples from Liñares Cave (38,000 BP, Northern Iberia) and Holocene samples from Denmark and modern *Cervus elaphus* (represented by the Polish WEST1 and WEST2). All these separate from another clade only constituted by some other Liñares Cave individuals, as previously described in [[5]](https://paperpile.com/c/i20Pps/VFwHW). These results suggest that the here reported sequences cervid belong to the western lineage that split from the eastern lineage one million years ago [[4]](https://paperpile.com/c/i20Pps/Q7VrE), belonging to the dominant clade encompassing European red-deer diversity (fig. SI4).

**Figure SI4**. Maximum likelihood tree of the Cervidae mtDNA genomes. The tree is rooted with *R. tarandus* mtDNA genomes. Colours denote clades. Green represents Western Clade A; Violet represents the Eastern *Cervus* diversity; red represents Western Clade B; orange Western Clade C; dark blue Western Clade D and light blue Western Clade E, according to Mackiewicz et al 2023 [[4]](https://paperpile.com/c/i20Pps/Q7VrE). All the sequences from Iberia bone tools are related to the rest of Clade A's diversity. These also show the genetic proximity of the Iberian bone tools to the Pleistocene sequences from Liñares cave in Spain. Numbers in the nodes depict bootstrap values.


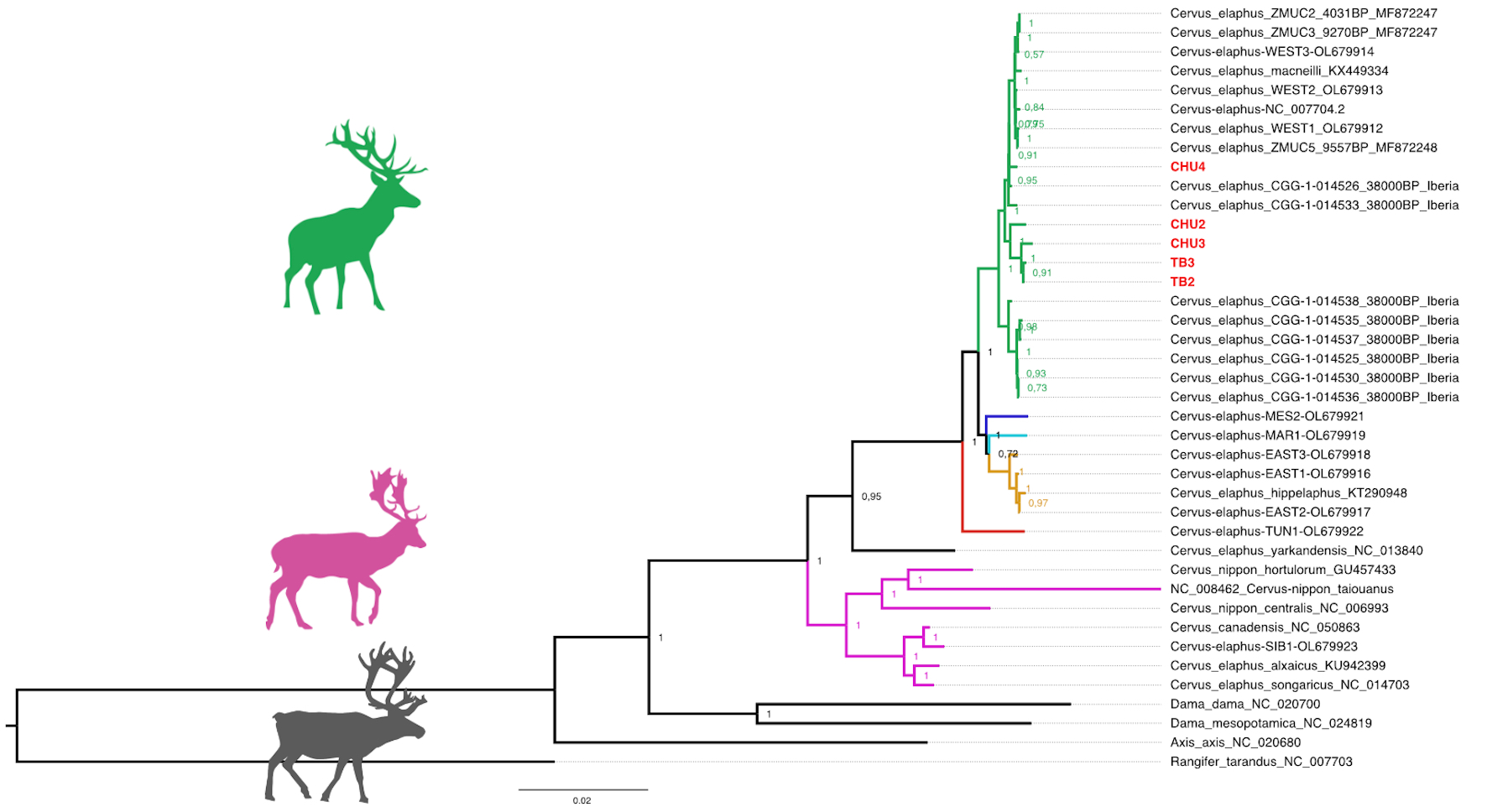


**Table SI1: List of species included in the in-solution hybridisation capture**

|  |
| --- |
| **Species** |
| *Alces alces* |
| *Apodemus sylvaticus* |
| *Arvicola amphibious* |
| *Bison bonasus* |
| *Bos taurus* |
| *Canis lupus* |
| *Capra hircus* |
| *Capreolus capreolus* |
| *Castor fiber* |
| *Cervus elaphus* |
| *Cricetus cricetus* |
| *Crocidura russula* |
| *Dama mesopotamica* |
| *Dicrostonyx hudsonius* |
| *Elephas maximus* |
| *Eliomys quercinus* |
| *Ellobius talpinus* |
| *Equus caballus* |
| *Erinaceus europaeus* |
| *Felis catus* |
| *Galemys pyrenaicus* |
| *Gazella subgutturosa* |
| *Homo sapiens* |
| *Hyaena hyaena* |
| *Hystrix brachyura* |
| *Lepus europaeus* |
| *Lynx lynx* |
| *Marmota marmota marmota* |
| *Martes martes* |
| *Meles meles* |
| *Micromys minutus* |
| *Microtus arvalis* |
| *Microtus subterraneus* |
| *Mus musculus* |
| *Mustela erminea* |
| *Mustela putorius* |
| *Neomis Fodiens* |
| *Neomys fodiensis* |
| *Oryctolagus cuniculus* |
| *Ovis aries* |
| *Panthera pardus* |
| *Rangifer tarandus* |
| *Rattus norvegicus* |
| *Rhinoceros unicornis* |
| *Rubicapra rubicapra* |
| *Saiga tatarica* |
| *Sorex araneus* |
| *Stylodipus telum* |
| *Sus scrofa* |
| *Talpa europaea* |
| *Ursus arctos* |
| *Vulpes vulpes* |

**References list**

[[1]](https://paperpile.com/c/i20Pps/tbkRV) Averbouh A., Téchnologie de la matière osseuse travaillée et implications palethnologiques. L’exemple des chaines d’exploitation du bois de cervidé chez les Magdaleniens des Pyrénées, Préhistoire-Ethnologie-Anthropologie, PhD, Université Paris I Panthéon-Sorbonne, 2000.

[[2]](https://paperpile.com/c/i20Pps/tbkRV) Averbouh A., Methodological specificities of techno-economic analysis of worked bone and antler: mental refitting and methods of application Crafting Bone: Skeletal Technologies through Time and Space: Proceedings of the 2nd Meeting of the (ICAZ) Worked Bone Research Group, Budapest, 31 August - 5 September 1999, B.A.R. 2001 111-121.

[[3]](https://paperpile.com/c/i20Pps/tbkRV) Korneliussen T.S., Albrechtsen A., Nielsen R., ANGSD: analysis of next generation sequencing data, BMC Bioinf. 15 (2014) 356.

[[4]](https://paperpile.com/c/i20Pps/Q7VrE)Mackiewicz P., Matosiuk M., Świsłocka M., Zachos F.E., Hajji G.M., Saveljev A.P., Seryodkin I.V., Farahvash T., Rezaei H.R., Torshizi R.V., Mattioli S., Ratkiewicz M.,

Phylogeny and evolution of the genus *Cervus* (Cervidae, Mammalia) as revealed by complete mitochondrial genomes, Sci. Rep. 12 (2022) 16381.

[[5]](https://paperpile.com/c/i20Pps/Q7VrE) Rey-Iglesia A., Grandal-d’Anglade A., Campos P.F., Hansen A.J., Mitochondrial DNA of pre-last glacial maximum red deer from NW Spain suggests a more complex phylogeographical history for the species Ecol. Evol. 2017 10690-10700.

[[6]](https://paperpile.com/c/i20Pps/Q7VrE) Hassanin A., Delsuc F., Ropiquet A., Hammer C., Jansen van Vuuren B., Matthee C., Ruiz-Garcia M., Catzeflis F., Areskoug V., Nguyen T.T., Couloux A., Pattern and timing of diversification of Cetartiodactyla (Mammalia, Laurasiatheria), as revealed by a comprehensive analysis of mitochondrial genomes C. R. Biol. 2012 32-50.

[[7]](https://paperpile.com/c/i20Pps/Q7VrE) Kim H.-J., Hwang J.-Y., Park K.-J., Park H.-C., Kang H.-E., Park J., Sohn H.-J., The complete mitochondrial genome of Cervus canadensis (Erxleben, 1777), as a model species of Chronic Wasting Disease (CWD) Mitochondrial DNA B Resour 2020 2621-2623.

[[8]](https://paperpile.com/c/i20Pps/Q7VrE) Li Y., Ba H., Yang F., Complete mitochondrial genome of *Cervus elaphus songaricus* (Cetartiodactyla: cervinae) and a phylogenetic analysis with related species Mitochondrial DNA A DNA Mapp Seq Anal 2016 620-621.

[[9]](https://paperpile.com/c/i20Pps/Q7VrE) Wada K., Okumura K., Nishibori M., Kikkawa Y., Yokohama M., The complete mitochondrial genome of the domestic red deer (*Cervus elaphus*) of New Zealand and its phylogenic position within the family Cervidae Anim. Sci. J. 2010 551-557.

[[10]](https://paperpile.com/c/i20Pps/Q7VrE) Wada K., Nishibori M., Yokohama M., The complete nucleotide sequence of mitochondrial genome in the Japanese Sika deer (*Cervus nippon*), and a phylogenetic analysis between Cervidae and Bovidae Small Rumin. Res. 2007 46-54.
